# The impact of delayed mobilization on post-discharge outcomes after emergency abdominal surgery: A prospective cohort study in older patients

**DOI:** 10.1371/journal.pone.0241554

**Published:** 2020-11-06

**Authors:** Jenelle L. Pederson, Raj S. Padwal, Lindsey M. Warkentin, Jayna M. Holroyd-Leduc, Adrian Wagg, Rachel G. Khadaroo

**Affiliations:** 1 Department of Surgery, Faculty of Medicine & Dentistry, University of Alberta, Edmonton, Canada; 2 Division of General Internal Medicine, Faculty of Medicine & Dentistry, University of Alberta, Edmonton, Canada; 3 Alberta Diabetes Institute, Edmonton, Alberta, Canada; 4 Faculty of Medicine, University of Calgary, Calgary, Alberta, Canada; 5 Division of Geriatric Medicine, Department of Medicine, Faculty of Medicine & Dentistry, University of Alberta, Edmonton, Canada; 6 Department of Critical Care Medicine, Faculty of Medicine & Dentistry, University of Alberta, Edmonton, Canada; Technion - Israel Institute of Technology, ISRAEL

## Abstract

Surgeons are increasingly treating seniors with complex care needs who are at high-risk of readmission and functional decline. Yet, the prognostic importance of post-operative mobilization in older surgical patients is under-investigated and remains unclear. Thus, we evaluated the relationship between post-operative mobilization and events after hospital discharge in older people. Overall, 306 survivors of emergency abdominal surgery aged ≥65y who required help with <3 activities of daily living were prospectively followed at two Canadian tertiary-care hospitals. Time until mobilization after surgery was attained from hospital charts and *a priori* defined as ‘delayed’ (≥36h) or ‘early’ (<36h). Primary outcomes for 30-day and 6-month all-cause readmission/death after discharge were assessed in multivariable logistic regression. Patients had a mean age of 76 ± 7.7 years, 45% were women, 41% were ‘vulnerable-to-moderately-frail’, according to the Clinical Frailty Scale. Most common reasons for admission were gallstones (23%), intestinal obstructions (21%), and hernia (17%). Median time to post-operative mobilization was 19h (interquartile range 9−35); 74 (24%) patients had delayed mobilization. Delayed mobilization was independently associated with higher risk of 30-day readmission/death (19 [26%] *vs*. 22 [10%], P<0.001; adjusted odds ratio [aOR] 2.24, 95%CI 0.99–5.06, P = 0.05), but this was not statistically significant at 6-months (38 [51%] *vs*. 64 [28%], P<0.001; aOR 1.72, 95%CI 0.91−3.25, P = 0.1). One-quarter of older surgical patients stayed in bed for 1.5 days post-operatively. Delayed mobilization was associated with increased risk of short-term readmission/death. As older, more frail patients undergo surgery, mobilization of older surgical patients remains an understudied post-operative factor.

**Trial registration:** clinicaltrials.gov identifier: NCT02233153

## Introduction

Immobilization is potentially harmful. Adverse physiological changes, such as muscle atrophy and reduced limb strength and aerobic capacity [1], have been observed following immobilization. Immobilization has been associated with increased risk of disuse osteoporosis [2], cardiovascular complications [3], and death [3].

Consequently, one might expect early mobilization to be of benefit in improving surgical recovery. Early mobilization after surgery prevents prolonged bed rest and may potentially prevent surgical complications. Prior studies of mobilization in the adult population have shown reductions in length of stay by 1–3 days [3, 4] and lower rates of pulmonary embolism, urinary tract infection, and delirium [3, 5].

However, few published data in older people are available. Of 8 prior studies to examine the effect of early mobilization following abdominal and thoracic surgery, none were conducted in older cohorts [6]. Yet, there are reasons to specifically encourage early mobilization in older patients. In this patient population, early mobilization is seldom performed or immobility is under-recognized in hospital [7–9], which results in older patients remaining immobile longer than their younger counterparts [9]. Older patients often present with comorbidity and frailty and may be particularly susceptible to complications and functional decline, resulting from delayed mobilization [3]. Indeed, immobilization in hospitalized older people has been considered a potentially modifiable risk-factor in ‘the cascade to dependency’ and progression toward institutional care and death [10].

No less important, the number of older emergency surgical patients is increasing. Older-aged patients frequently undergo gastrointestinal surgery [11, 12], which represents a large proportion of inpatient surgery in Canada–twice that of hip replacement and coronary artery angioplasty in 2012 [13], and the highest rate of 30-day surgical readmission, with one of six ostomy patients readmitted [11]. Thus, we examined whether delayed versus early post-operative mobilization was associated with all-cause readmission or death, functional status, and quality-of-life after discharge in an older emergency abdominal surgery population.

## Methods

### Population and setting

We included patients who were prospectively enrolled during the pre-implementation phase of the Elder-Friendly Approaches to the Surgical Environment (EASE) study (clinicaltrials.gov identifier: NCT02233153) [14], a controlled before-and-after quality improvement study. Patients aged 65 years or older who underwent emergency abdominal surgery were recruited between January 2014 to September 2015 from general surgery services at two tertiary-care teaching hospitals in Alberta, Canada (University of Alberta Hospital and Foothills Medical Centre). Patients were excluded if they had baseline functional dependency (i.e., required help with ≥3 basic activities of daily living pre-operatively, with more likely inability to respond to the greater EASE intervention), underwent surgery that was for elective (i.e. surgery scheduled in advance as it does not involved a medical emergency, trauma or palliative reasons, or were transferred from another province, hospital or inpatient service. The University of Alberta Research Ethics Board (Pro00047180) and the University of Calgary Research Ethics Board (REB140729) approved study procedures.

### Mobilization following surgery

Information on time of mobilization following surgery was retrieved from detailed nursing care records. Mobilization was broadly defined as the first recorded instance out of bed after the initial surgery, which has been considered a minimum target for mobilization [15]. Specifically, “out of bed” corresponds to a range of ability to transfer from bed, but at the minimum, we used a definition of transferring from bed to a chair (or greater, such as ambulating in the hall) independently or with 2-person assistance. In absence of a widely-accepted, evidence-based threshold for time of post-operative mobilization in older patients, mobilization was *a priori* defined as ‘delayed’ if ≥36 hours or ‘early’ if <36 hours following surgery, corresponding to the upper quartile limit for time of mobilization in this cohort.

### Outcomes

Primary outcomes were 30-day and 6-month all-cause readmission or death after discharge following surgery. Secondary outcomes were: 5-week patient-reported functional performance and independence (Edmonton Frail Scale [16]); 5-week Timed Up and Go Test (TUGT) [17]; and 5-week and 6-month health-related quality-of-life (EurolQoL health questionnaire [18] [five dimensions: EQ-5D, visual analogue scale: EQ-VAS]; 12-item Short-form Health Survey [19] [SF-12: mental and physical component]).

### Data collection and measurements

Baseline demographic and clinical characteristics were abstracted by trained research assistants through chart reviews and patient interviews in-hospital and at clinic follow-up. Covariates included the Charlson Comorbidity Index (range1−6); pre-admission frailty, assessed according to the Clinical Frailty Scale (CFS) [20] (dichotomized score≥4, ‘vulnerable-to-moderately-frail’ and score<4 ‘very-fit-to-managing-well’); the American Society of Anesthesiologists (ASA) Physical Status Classification (range I-VI); and surgical procedure (laparoscopic cholecystectomy or appendectomy, open cholecystectomy or appendectomy, hernia repair, small intestinal resection or lysis of adhesions, colectomy, and other). Of note, ‘very fit to managing well’ encompasses patients considered ‘very fit’ (score 1) as in the most active and energetic, ‘well’ (score 2) with occasional activity, and ‘managing well’ (score 3) not physically active beyond routine walking, while the “vulnerable–moderately frail’ group includes patients considered ‘vulnerable’ (score 4) with limited activities due to comorbidities but no disability, ‘mildly frail’ (score 5) with dependence in ≥ 1 instrumental activity of daily living (e.g., cooking, housework), and ‘moderately frail’ (score 6) with dependence in 1 or 2 basic activities of daily living (e.g. bathing, dressing).

Outcomes for readmission and mortality were collected from a province-wide electronic medical records database. Secondary outcomes were ascertained through in-person or telephone follow-up at 5 weeks after discharge and by telephone at 6 months. The TUGT mobility assessment was considered as time to rise from a chair, walk 10ft, and return to sitting; we considered ≥14secs, the upper quartile limit, as abnormal (also defines fall risk [21]). Functional performance was considered reduced if patients reported that they were unable to walk 1km without help and unable to walk up one flight of stairs without help [16]; functional independence was considered reduced if patients reported dependence in >1 instrumental activity of daily living [IADLs] [16]. The EQ-5D assessed domains of perceived mobility, self-care, usual activities, pain/discomfort, and anxiety/depression, where lower scores indicate worse health-related quality-of-life and a minimal clinically important difference (MCID) was 0.03-points [22]. The EQ-VAS assessed self-rated health state (0 ‘worst’ to 100 ‘best’; MCID = 10.0 [22]). The SF-12 assessed physical and mental components of health-related quality-of-life, with lower scores indicating worse health status (MCID = 5.0 [23]). Health-related quality-of-life scores for both groups of delayed and early mobilization were compared against age-specific norms, specifically Alberta population normative data for the EQ-5D and Canadian population normative data for the SF-36 [24, 25].

### Statistical analysis

Descriptive statistics were calculated, including means, medians and proportions. First, we identified potentially confounding variables by literature review and bivariate analyses, comparing baseline characteristics, including a wide range of patient and perioperative factors, by outcomes and delayed versus early mobilization with a predefined cut-off of p-value <0.2 (statistical tests indicated in tables). Second, multivariable logistic regression models were fit to calculate the adjusted odds of 30-day and 6-month readmission or death for patients with delayed versus early mobilization. Potential confounders that were assessed in the model are listed in tables. Age and sex were retained in all models. Other covariates were retained in the model if they met statistical criteria (bivariate p-value <0.1 or >10% change in beta-coefficient for mobilization on inclusion). The most parsimonious models were selected. All covariates were assessed for collinearity by calculating Pearson’s correlation coefficient. Third, differences between patients with delayed versus early mobilization in functional status (defined as binary variables) were assessed using multivariable logistic regression after adjusting for sex, frailty, and surgery type. Fourth, t-tests were used to compare health-related quality-of-life scores between delayed versus early mobilization groups and between both groups versus age-specific normative population data [24, 25]. Finally, sensitivity analyses were *a prior* planned and conducted to examine the relationship between delayed mobilization and frailty (CFS≥4) by (1) stratifying the adjusted-relationship by frailty status and (2) assessing interaction terms in the main effects models on multiplicative and additive scales. To estimate additive interaction, we calculated the relative excess risk due to interaction (RERI_OR_) from the adjusted odds ratios [26]. We assessed model fit by Hosmer-Lemeshow goodness-of-fit tests and accuracy by the C-statistic with 95% confidence intervals (95%CI). Moreover, we assessed models that excluded patients requiring open cholecystectomies, open appendectomies, and laparotomies; a separate analysis also patients requiring 2 or more surgeries. Lastly, time of mobilization was secondarily categorized by 12 hours to allow sufficient number of patients in each category for analysis and based on the above cut-off (>48h n = 47, 48-36h n = 27, 35-24h n = 31, 24-12h n = 102, <12h n = 99). Statistical significance was defined as a two-tailed p-value <0.05. All analyses were conducted in STATA 13 (StataCorp LP, College Station, TX, USA, 2013).

## Results

**Fig 1:**
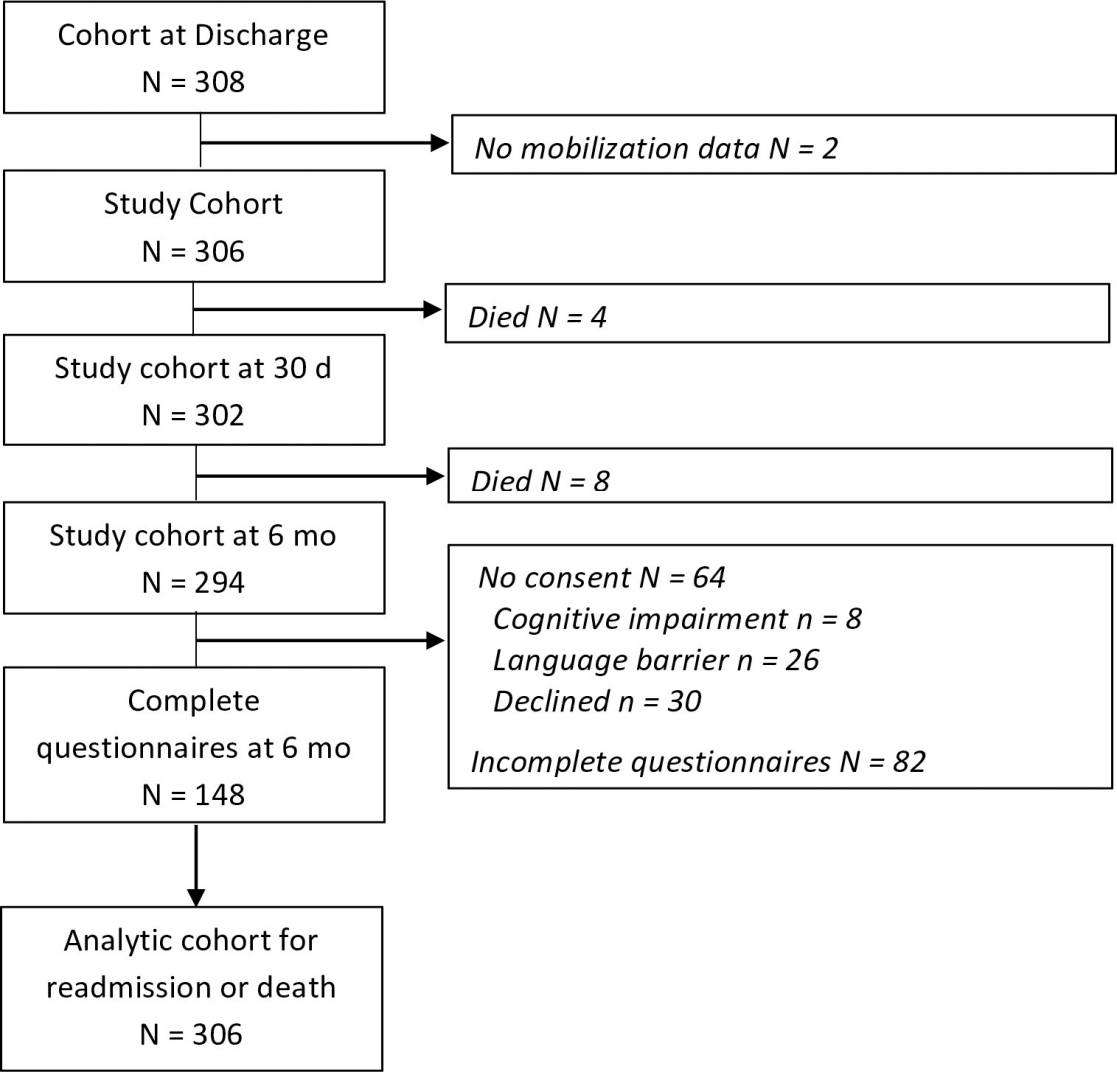
Flow diagram of cohort.

Altogether, 3506 admissions were screened; major exclusions were age <65 years (70%) and conservative management (9%) [27]. A total of 322 patients were included; 14 patients died in-hospital and 2 patients had missing mobilization data, thus excluding 16 patients from primary analyses (Fig 1). Complete outcome assessment was achieved for 30-day and 6-month readmission and mortality. Of enrolled patients able to answer questionnaires (i.e., English speaking), 148 (64%) completed 6-month interviews. Patients with and without completed 6-month questionnaires were comparable in demographics, medications, Charlson Comorbidity Index, ASA class, surgery type, length of stay, and time of mobilization, but were slightly less frail and most were Caucasian (online-only S1 Table).

Overall, 306 patients were included in the final analysis. Mean age was 76 ± 7.7 years (range 65−97), 138 (45%) were women, and 126 (41%) had a frailty status of ‘vulnerable-to-moderately-frail’. No patients were severely frail (>6 CFS), as those patients did not meet study inclusion. The median Charlson Comorbidity Index score was 1 (interquartile range [IQR] 0−2) and the median ASA Class was 3 (IQR 2−3). A total of 69 (23%) patients were admitted for gallstones, 65 (21%) for intestinal obstruction, 52 (17%) for hernia procedures, 36 for appendicitis, and 22 for cancer/tumors. Specifically, 15 (5%) patients underwent ventral hernia repair, 23 (8%) patients underwent inguinal hernia repair, and 6 (2%) patients underwent both ventral and inguinal hernia repair of a total 306 surgeries (Table 1). Fourteen patients required 2 surgeries and 2 patients required >2 (Table 1). Eleven (4%) patients received geriatric medicine consultation and 23 were referred for acute pain care.

**Table 1:** Characteristics of older emergency abdominal surgery patients, by time of mobilization.

Characteristic	Delayed Mobilization (≥36 h)	Early Mobilization (<36 h)	P value^a^
	N = 74 (%)	N = 232 (%)	
Age, years^c^^,^^d^			0.7
65–75	34 (46)	119 (51)	
75–85	29 (39)	78 (34)	
>85	11 (15)	35 (15)	
Sex, female^c^^,^^d^	41 (55)	97 (42)	0.04
Married^c^	42 (57)	168 (72)	0.01
Prior living situation^c^			<0.001
Home without assistance	41 (55)	186 (80)	
Home with assistance	22 (30)	42 (18)	
Supportive facility	9 (12)	4 (2)	
Patients with at least one prior hospitalization^c^	19 (26)	53 (23)	0.6
Charlson Comorbidity Index, median (IQR)^c^^,^^d^	1 (1–2)	1 (0–1)	<0.001^b^
Patients in abnormal range at admission			
Hemoglobin, men (<140g/L or >185g/L)^c^^,^^d^	21 (28)	52 (22)	0.009
Hemoglobin, women (<123g/L or >165g/L)^c^^,^^d^	12 (16)	34 (15)	0.5
Sodium (<135mmol/L or >150mmol/L)^c^	20 (27)	49 (21)	0.3
Total admission medications, median (IQR)^c^^,^^d^	6 (4−8)	3 (2−6)	<0.001^b^
Clinical Frailty Scale, mean ± SD^c^^,^^d^	4.4 ± 1.4	3.2 ± 1.2	<0.001
ASA Physical Status Class, mean ± SD^c^	3.3 ± 0.8	2.5 ± 0.7	<0.001
Post-operative disposition			<0.001
Ward	42 (57)	226 (98)	
Close observation	2 (3)	3 (1)	
ICU	30 (40)	3 (1)	
Surgery Type^c^			<0.001
Closed appendectomy or cholecystectomy	1 (1)	85 (37)	
Open appendectomy or cholecystectomy	6 (8)	17 (7)	
Hernia	6 (8)	36 (16)	
Small intestine	31 (42)	55 (24)	
Colon	20 (27)	23 (10)	
Other	10 (14)	16 (7)	
Colostomy or ileostomy creation procedure	17 (23)	11 (5)	<0.001
Patients requiring > 2 procedures	10 (14)	6 (3)	<0.001
Time under anesthesia (hours)^d^	2.3	1.7	<0.001
Consults			
Geriatrics	9 (12)	2 (0.9)	<0.001
Acute Pain Service	10 (14)	12 (5)	0.02
Length of stay to discharge, median (IQR)^c^	14 (10−28)	7 (5−11)	<0.001^b^

Note. ASA = American Society of Anesthesiologists; SD = standard deviation, IQR = interquartile range.

^a^*X*^2^ or Fischer’s exact tests for categorical and *t*-test for continuous variables unless otherwise specified.

^b^Mann-Whitney test.

^c^Potential confounders in bivariate analysis that were assessed in models. Additional variables potential confounders that were assessed in models not included above: admission source (such as ED, transfer, ICU), creatinine on admission, post-operative disposition (ward, PACU, ICU), major and minor post-operative complications, use of TPN after surgery, discharge status, and most responsible diagnosis)

^d^Variables included as covariates in models.

### Delayed mobilization

The median time of mobilization following surgery was 19h (IQR 9−35). Delayed mobilization was present in 74 (24%) patients. Patients were similar in age and the number with prior hospitalization, but those with delayed mobilization were slightly more frail, had higher total medications, and more required assistance at home (Table 1). Patients with delayed mobilization had somewhat higher ASA class scores and were more likely to receive specialized geriatric medicine or pain care (Table 1).

### Readmission or death

**Fig 2:**
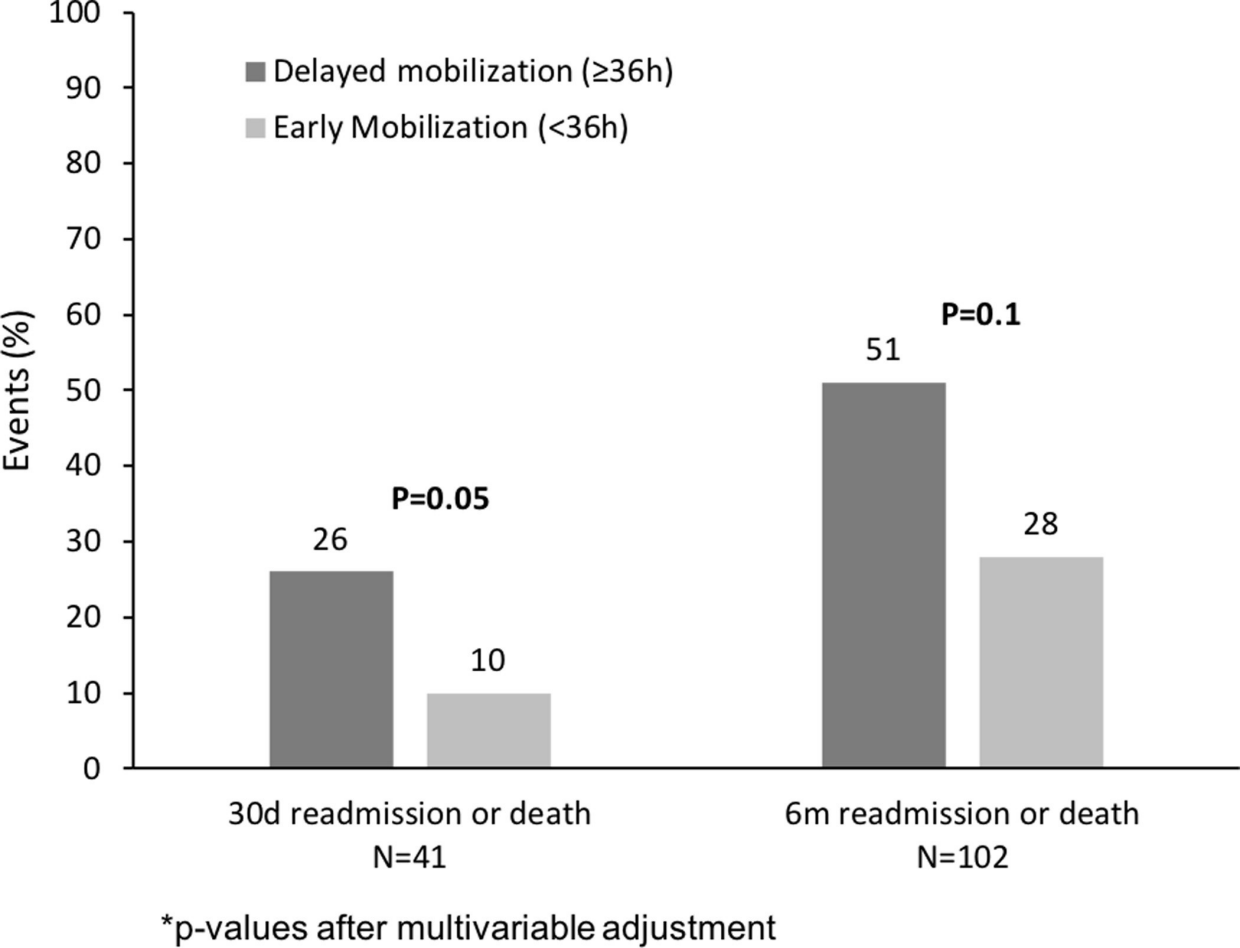
Readmission or death after surgical discharge, according to time of mobilization.

Overall, 41 of 306 patients were readmitted or died within 30 days after discharge, increasing to 102 patients by 6 months. Patients with delayed mobilization had higher incidence of 30-day readmission or death (19/74 [26%] *vs*. 22/232 [10%] P<0.001) and 6-month readmission or death (38/74 [51%] *vs*. 64/232 [28%] P<0.001; Fig 2 and Table 2), compared to early mobilization. After adjusting for age, sex, Charlson Comorbidity Index, time under anesthesia, number of medications, hemoglobin, and frailty, delayed mobilization was associated with 2.2-times greater risk of 30-day readmission or death (adjusted odds ratio [aOR] 2.24, 95%CI 0.99−5.06, P = 0.05; C-statistic = 0.75, 95%CI 0.66−0.84; Table 2). There was no statistically significant increase in 6-month risk (aOR 1.72, 95%CI 0.91−3.25, P = 0.1; C-statistic = 0.73, 95%CI 0.67−0.79; Table 2). Readmissions accounted for most events (Table 2). Twelve patients died within 6 months of discharge; patients with delayed rather than early mobilization more frequently died after 30-days (3/74 [4%] *vs*. 1/232 [0.4%], P = 0.04), but not 6-months (6/74 [7%] *vs*. 6/232 [3%], P = 0.09) (Table 2).

**Table 2:** Outcomes after surgical discharge, according to time of mobilization.

	No. of patients in mobilization group (%)			Relationship of delayed mobilization and outcome			
Outcome	Delayed (≥36 h)	Early (<36 h)	P value†	Crude OR (95%CI)	P value	aOR (95%CI)	P value
Clinical status							
*30 days*	*N = 74*	*N = 232*					
Readmission/death	19 (26)	22 (10)	<0.001	3.30 (1.67−6.52)	0.001	2.24 (0.99−5.06)	0.05
Readmission alone	18 (24)	22 (10)	0.001	3.07 (1.54−6.11)	0.001	2.11 (0.93−4.81)	0.08
*6 months*	*N = 74*	*N = 232*					
Readmission/death	38 (51)	64 (28)	<0.001	2.77 (1.62−4.75)	< 0.001	1.71 (0.91−3.25)	0.1
Readmission alone	35 (47)	63 (27)	0.001	2.41 (1.40−4.13)	0.001	1.57 (0.84−2.96)	0.2
Functional status							
*5 weeks*	*N = 37*	*N = 120*					
Unable to walk 1km without help^a^	25 (68)	35 (29)	<0.001	5.06 (2.29−11.18)	< 0.001	3.23 (1.35−7.75)	0.009
Unable to walk flight of stairs without help ^a^	17 (46)	20 (17)	<0.001	4.25 (1.90−9.51)	< 0.001	2.54 (1.04−6.23)	0.04
Dependent in >1 IADL^b^^,^ ^c^	23 (62)	25 (21)	<0.001	6.18 (2.78−13.71)	<0.001	3.83 (1.57−9.35)	0.003
Abnormal gait^d^	9 (41)	17 (23)	0.1	2.31 (0.85−6.34)	0.1	1.63 (0.47−5.65)	0.4

Note. IADLs = instrumental activities of daily living; TUGT = timed up and go test; aOR = adjusted odds ratio; CI = confidence interval; IQR = interquartile range.

Multivariable logistic regression models for clinical outcomes adjusted for age, sex, comorbidities, time under anesthesia, total medications, abnormal hemoglobin, and frailty. The 6-month model was additionally adjusted for ostomy creation procedure.

Multivariable logistic regression models for functional outcomes adjusted for frailty, sex, and type of surgery.

All models had a Hosmer-Lemeshow goodness-of-fit with *P*>0.1 and c-statistic>0.7.

^a^Patient-reported dependence to complete activities as assessed by the Edmonton Frail Scale [16].

^b^*X*^2^ tests for categorical and Mann-Whitney test for continuous variables.

^c^In early mobilization group, 119 completed follow-up questionnaires.

^d^ In 22 delayed and 74 early to mobilize who attended surgery follow-up appointment.

### Functional status

At 5-weeks, over one-quarter of patients reported reduced functional performance (60 [38%] patients unable to walk 1km; 37 [24%] unable to walk stairs) and diminished independence (48 [31%] dependent in >1 IADLs) and 26 (27%) patients had abnormal TUGT (median 11s, IQR 9−14). Patients with delayed rather than early mobilization had reduced functional performance and independence, but did not have abnormal TUGT after adjusting for sex, frailty, and surgery type (Table 2).

### Health-related quality-of-life

Patients with delayed mobilization reported lower 5-week overall health-related quality-of-life, compared to patients with early mobilization, which was clinically important according to the EQ-5D and EQ-VAS MCIDs (Table 3). Moreover, the delayed mobilization group had lower 5-week EQ-5D and EQ-VAS scores than the general population while the early group did not (Table 3). Patients with delayed mobilization also reported lower 5-week physical, but not mental, health-related quality-of-life according to the SF-12. Five-week differences in the SF-12 were generally not considered clinically important and both groups had lower SF-12 scores than the general population (Table 3). Overall, health-related quality-of-life improved from 5 weeks to 6 months after discharge in patients with delayed mobilization, but were relatively consistent and unchanged in the early group (online-only S2 Table).

**Table 3:** Health-related quality-of-life at 5-weeks and 6-months.

	Time of mobilization, mean ± SD		Difference between groups, mean (p-value)		
Measure	Delayed (>35 h)	Early (≤35 h)	Delayed *vs*. early	Delayed *vs*. population†	Early *vs*. population†
*5 weeks*	*N = 35*	*N = 118*			
EQ-5D	0.73 ± 0.2, n = 36	0.86 ± 0.16	-0.13* (< 0.001)	-0.11* (< 0.001)	0.02 (0.2)
EQ-VAS	68.0 ± 18.9	78.5 ± 14.7, n = 119	-10.5* (< 0.001)	-9.8 (0.001)	0.7 (0.7)
SF-12, physical	36.7 ± 6.3	40.2 ± 5.9	-3.5 (0.003)	-8.7* (< 0.001)	-5.1* (< 0.001)
SF-12, mental	51.6 ± 8.3	52.1 ± 6.7	-0.5 (0.7)	-2.4 (0.09)	-1.9 (0.02)
*6 months*	*N = 39*	*N = 109*			
EQ-5D	0.79 ± 0.23	0.88 ± 0.16, n = 107	-0.09* (0.01)	-0.05* (0.06)	0.04* (0.02)
EQ-VAS	70.5 ± 18.5	76.7 ± 18.1	-6.2 (0.07)	-7.3 (0.009)	-1.1 (0.6)
SF-12, physical	39.4 ± 6.6	41.0 ± 5.5	-1.6 (0.1)	-6.0* (< 0.001)	-4.4 (< 0.001)
SF-12, mental	52.9 ± 6.1	52.9 ± 5.6	0.0 (>0.99)	-1.1 (0.4)	-1.1(0.2)

Notes: EQ-5D = EurolQol five dimension index score; EQ-VAS = visual analogue scale; SF-12 = 12-item Short-form Health Survey

*Minimal clinically important difference (EQ-5D = 0.03; EQ-VAS = 10.0; SF-12 = 5.0)

†Normative EQ data for 251 individuals in Alberta [24] and normative SF-12 data for 4513 individuals in Canada [25] aged ≥65 years randomly sampled from the general population.

### Sensitivity analysis

In stratified analyses by frailty status, delayed mobilization independently predicted 30-day readmission or death (aOR 3.39, 95%CI 1.22−9.38, P = 0.02) and 6-month readmission or death (aOR 2.74, 95%CI 1.08−6.93, P = 0.03) among ‘vulnerable-to-moderately-frail’ patients (CFS≥4, n = 126), but not ‘very-fit-to-managing-well’ patients (CFS<4, n = 180, 30-day: aOR 2.50, 95%CI 0.68−9.14, P = 0.2 and 6-month: aOR 1.45, 95%CI 0.53−3.95, P = 0.5; online-only S3 Table). We did not observe statistically significant interaction between delayed mobilization and frailty for readmission or death on the multiplicative scale (30-day: aOR 0.99, 95%CI 0.20–4.94, P≥0.99 and 6-month: aOR 1.45, 95%CI 0.40−5.20, P = 0.6) or additive scale [30-day: RERI_OR_ 1.68, 95%CI -3.29−6.65, P = 0.5 and 6-month: RERI_OR_ 1.58 95%CI -1.40−4.55, P = 0.3, online-only S3 Table]. The adjusted relationship between the time of mobilization (based on every 12 hrs) and readmission or death did not appear to follow a dose response (data available on request). Restricting the 30-day model to patients with only 1 surgery, there was no significant change: aOR 2.38, 95%CI 0.99–5.73, p = 0.05 and 6-month: aOR 1.58, 95%CI 0.79–3.16, p = 0.2. Frequency of events based on time of mobilization are included online (online-only S4 Table).

## Discussion

### Main findings

One-quarter of older abdominal surgery patients were immobile for at least 1.5 days following surgery. Patients with delayed mobilization had over 2-times greater adjusted risk of 30-day readmission or death after discharge, compared to early mobilization; this increased risk was not statistically significant at 6 months. The observed association was stronger in frail patients, with up to 3-fold increased risk of readmission or death for patients with delayed mobilization in this vulnerable subgroup. Five-weeks post-discharge, delayed mobilization was associated with lower patient-reported functional performance and independence, irrespective of sex, frailty, and surgery type, and lower patient-reported overall health status, as well as physical health-related quality-of-life.

We found that delayed mobilization in older abdominal surgery patients was associated with poor short-term outcomes. Prior evidence on the impact of mobilization has been lacking, owing to scarce data on older people and outcomes after hospital discharge [3, 6]. Moreover, most comparable studies have been conducted in orthopedic populations for which surgery directly affects mobility [3, 5, 28]. Our findings differ from a prior feasibility study in 116 adults undergoing elective gastrointestinal surgery that found no difference in 30-day readmission rates, comparing early post-operative mobilization to usual care (<24 h) [29]. Similarly, results from a randomized control trial of 200 adult surgical ICU patients mechanically ventilated for less than 48 hours found no difference in 3-month post-discharge mortality with early goal-directed mobilization [30]. Yet, in a study of 532 hip fracture patients, patients with baseline frailty similarly had the highest adjusted risk of 30-day mortality associated with longer immobilization [28].

Multiple mechanisms could explain our observations. First, physiological changes like reduced muscle mass or strength during periods of immobility [1] could impede recovery of baseline function or result in hospitalization-associated disability [8] even after resolution of surgical issues, which could have more profound impacts in adults with frailty. Second, in-hospital immobilization may impair cognition or processes of pain, eating, or inflammation [10], potentially increasing susceptibility to post-hospital syndrome, a transient condition of depleted physiological reserve [31]. This might explain higher observed 30-day, and not 6-month, risk of events, and heighted risk in frail groups. Delayed mobilization could lead to complications, a common reason for 30-day surgical readmission [11], or comorbid disease exacerbations that often require hospitalization [11]. Residual confounding could partly explain the association if patients with delayed mobilization also had more comorbidity, frailty, or complicated surgery or surgical course prior to mobilization, and these latter factors, not delayed mobilization, resulted in readmission. However, the observed association remained in our study, as well as prior studies [3, 5, 6, 28], after adjusting for age, hemoglobin, baseline frailty, comorbidities, and time under anesthesia. Neither additionally controlling for surgery type nor post-operative complications appreciably changed results (data available in supplement)

### Limitations

There are multiple limitations to this study. First, we did not assess for the quantity of time out of bed or active mobilization and thus, cannot determine a dose-response relationship between physical activity and prognosis or assess the impact of different types of mobilization. However, time to mobilization out of bed is a relevant target used elsewhere [28] that requires relatively few in-hospital resources. Second, the observational nature of this study mandates that findings be viewed as associative, not causative, in nature. Residual confounding is a limitation of the prospective observational study design. In particular, the cohort is heterogeneous, including patients who underwent a variety of surgeries. Thus, our model was adjusted for time under anesthesia, which may account for differences in type or difficulty of surgery. Third, as we did not measure function or health-related quality-of-life in patients prior to their surgery, we could not examine changes from baseline. Fourth, we had limited information on the supportive measures to optimize older patients prior to surgery, which may have an important role on improving surgical outcomes. Finally, we were unable to assess for differences based on open versus closed procedures.

Additional studies are needed to define mobilization protocols specific to older people, which might also assess barriers to mobilization (e.g., lack of patient or care-provider confidence regarding safety, the false assumption that bed rest improves healing in older people, or physical barriers like catheter use). Particularly, randomized controlled trials are required to examine the short and long-term benefits and risks of mobilizing older patients earlier after abdominal surgery.

## Conclusion

Delayed mobilization in older abdominal surgery patients was common and associated with increased risk of adverse events and lower patient-reported outcomes shortly after discharge. Older patients with longer than expected periods of immobilization are a group to target for evidence-based interdisciplinary discharge and transition programs, particularly since post-operative complications often occur shortly after discharge and elderly patients are at high risk of early readmission. However, since delays in mobilization are also potentially modifiable and may intersect with other less amendable surgical risk factors like comorbidity or frailty, elder-specific strategies targeting early mobilization need to be evaluated.

## Supporting information

S1 Table Baseline characteristics, according to completion at 6-month follow-up. (DOCX)

S2 Table Changes in health-related quality-of-life from 5 weeks to 6 months after surgical discharge. (DOCX)

S3 Table Delayed mobilization and readmission or death, stratified by frailty status. (DOCX)

S4 Table Time to mobilization and readmission or death. (DOCX)

## References

[1] Kortebein P, Symons TB, Ferrando A, Paddon-jones D, Ronsen O, Protas E, et al Functional Impact of 10 Days of Bed Rest in Healthy Older Adults. J Gerontol A Biol Sci Med Sci. 2008;63(10):1076–81. 10.1093/gerona/63.10.1076 18948558

[2] Dittmer DK, Teasell R. Complications of immobilization and bed rest. Part 1: Musculoskeletal and cardiovascular complications. Can Fam Physician. 1993;39:1428–32, 1435–7. 8324411 PMC2379624

[3] Kalisch BJ, Lee S, Dabney BW. Outcomes of inpatient mobilization: A literature review. J Clin Nurs. 2014;23(11–12):1486–501. 10.1111/jocn.12315 24028657

[4] Pashikanti L, Von Ah D. Impact of Early Mobilization Protocol on the Medical-Surgical Inpatient Population. Clin Nurse Spec. 2012;26(2):87–94. 10.1097/NUR.0b013e31824590e6 22336934

[5] Epstein N. A review article on the benefits of early mobilization following spinal surgery and other medical/surgical procedures. Surg Neurol Int 2014;5:S66–73. 10.4103/2152-7806.130674 24843814 PMC4023009

[6] Castelino T, Fiore JF, Niculiseanu P, Landry T, Augustin B, Feldman LS. The effect of early mobilization protocols on postoperative outcomes following abdominal and thoracic surgery: A systematic review. Surgery. 2016;159(4):991–1003. 10.1016/j.surg.2015.11.029 26804821

[7] Kalisch BJ, Landstrom G, Williams RA. Missed nursing care: Errors of omission. Nurs Outlook. 2009;57(1):3–9. 10.1016/j.outlook.2008.05.007 19150261

[8] Covinsky KE, Pierluissi E, Johnston CB. Hospitalization-Associated Disability “She Was Probably Able to Ambulate, but I’m Not Sure.” JAMA. 2011;306(16):1782–93. 10.1001/jama.2011.1556 22028354

[9] Brown CJ, Redden DT, Flood KL, Allman RM. The underrecognized epidemic of low mobility during hospitalization of older adults. J Am Geriatr Soc. 2009;57(9):1660–5. 10.1111/j.1532-5415.2009.02393.x 19682121

[10] Creditor MC. Hazards of hospitalization of the elderly. Ann Intern Med. 1993;118(3):219–23. 10.7326/0003-4819-118-3-199302010-00011 8417639

[11] Canadian Institute for Health Information. All-Cause Readmission to Acute Care and Return to the Emergency Department (Ottawa, Ont.: CIHI, 2012).

[12] Deiner S, Westlake B, Dutton RP. Patterns of surgical care and complications in elderly adults. J Am Geriatr Soc. 2014;62(5):829–35. 10.1111/jgs.12794 24731176 PMC4024102

[13] Canadian Institute for Health Information. Hospital Morbidity Database (Ottawa, Ont.: CIHI, 2012–2013) Available from: https://www.cihi.ca/en/quick-stats

[14] Khadaroo RG, Padwal RS, Wagg AS, Clement F, Warkentin LM, Holroyd-Leduc J. Optimizing senior’s surgical care—Elder-friendly Approaches to the Surgical Environment (EASE) study: rationale and objectives. BMC Health Serv Res. 2015;15:338 10.1186/s12913-015-1001-2 26293153 PMC4546177

[15] Liu B, Almaawiy U, Moore JE, Chan W-H, Straus SE. Evaluation of a multisite educational intervention to improve mobilization of older patients in hospital: protocol for mobilization of vulnerable elders in Ontario (MOVE ON). Implement Sci. 2013;8(1):76 10.1186/1748-5908-8-76 23822563 PMC3704763

[16] Rolfson DB, Majumdar SR, Tsuyuki RT, Tahir A, Rockwood K. Validity and reliability of the Edmonton Frail Scale. Age Ageing. 2006;35(5):526–9. 10.1093/ageing/afl041 16757522 PMC5955195

[17] Podsiadlo D, Richardson S. The timed “Up & Go”: a test of basic functional mobility for frail elderly persons. J Am Geriatr Soc. 1991;39(2):142–8. 10.1111/j.1532-5415.1991.tb01616.x 1991946

[18] EurolQol Group. EQ-5D: A standardized instrument for use as a measure of health outcomes [EurolQol web site]. 1990. Available at: http://. Accessed March 27, 2017.

[19] SF-36.org. The SF-12(r): an even shorter health survey [Optum web site]. 1996. Available at http://www.sf-36.org/tools/sf12.shtml. Accessed March 27, 2017.

[20] Rockwood K, Song X, MacKnight C, Bergman H, Hogan DB, McDowell I, et al A global clinical measure of fitness and frailty in elderly people. CMAJ. 2005;173(5):489–95. 10.1503/cmaj.050051 16129869 PMC1188185

[21] Shumway-Cook A, Brauer S, Woollacott M. Predicting the probability for falls in community-dwelling older adults using the Timed Up & Go Test. Phys Ther. 2000;80(9):896–903. 10960937

[22] Luo N, Johnson JA, Coons SJ. Using instrument-defined health state transitions to estimate minimally important differences for four preference-based health-related quality of life instruments. Med Care. 2010;48(4):365–71. 10.1097/mlr.0b013e3181c162a2 20355266

[23] Wyrwich KW, Tierney WM, Babu AN, Kroenke K, Wolinsky FD. A comparison of clinically important differences in health-related quality of life for patients with chronic lung disease, asthma, or heart disease. Heal Serv Res. 2005;40(2):577–91. 10.1111/j.1475-6773.2005.00373.x 15762908 PMC1361158

[24] Johnson J, Pickard AS. Comparison of the EQ-5D and SF-12 health surveys in a general population survey in Alberta, Canada. Med Care. 2000;38(1):115–21. 10.1097/00005650-200001000-00013 10630726

[25] Hopman WM, Towheed T, Anastassiades T, Tenenhouse A, Poliquin S, Berger C, et al Canadian normative data for the SF-36 health survey. Canadian Multicentre Osteoporosis Study Research Group. CMAJ. 2000;163(3):265–71. 10951722 PMC80287

[26] Vanderweele TJ, Knol MJ. A Tutorial on Interaction. Epidemiol Methods. 2014;3(1):33–72. 10.1515/em-2013-0005

[27] Li Y, Pederson J, Churchill T, Wagg A, Holroyd-Leduc J, Alagiakrishnan K, et al Impact of frailty on outcomes after discharge in older surgical patients: a prospective cohort study. CMAJ. 2018;2 2(190):E184–90. 10.1503/cmaj.161403 29565018 PMC5828889

[28] Siu AL, Penrod JD, Boockvar KS, Koval K, Strauss E, Morrison RS. Early Ambulation After Hip Fracture: effects on function and mortality. Arch Intern Med. 2006;166(7):766–71. 10.1001/archinte.166.7.766 16606814 PMC3045760

[29] van der Leeden M, Huijsmans R, Geleijn E, de Lange-de Klerk ESM, Dekker J, Bonjer HJ, et al Early enforced mobilisation following surgery for gastrointestinal cancer: Feasibility and outcomes. Physiotherapy. 2016;102(1):103–10. 10.1016/j.physio.2015.03.3722 26059985

[30] Schaller SJ, Anstey M, Blobner M, Edrich T, Grabitz SD, Gradwohl-Matis I, et al Early, goal-directed mobilisation in the surgical intensive care unit: a randomised controlled trial. Lancet. 2016;(10052):1377–88. 10.1016/S0140-6736(16)31637-3 27707496

[31] Krumholz HM. Post-Hospital Syndrome—An Acquired, Transient Condition of Generalized Risk. N Engl J Med. 2013 1 10;368(2):100–2. 10.1056/NEJMp1212324 23301730 PMC3688067

