# Supplementary material for: The impact of delayed mobilization on post-discharge outcomes after emergency abdominal surgery: A prospective cohort study in older patients
Source: PLoS One. 2020 Nov 6;15(11):e0241554. doi: 10.1371/journal.pone.0241554 (PMC7647086; doi:10.1371/journal.pone.0241554)
Supplement: S1 Table — (DOCX) [file pone.0241554.s001.docx]

S1 Table: Baseline characteristics, according to completion at 6-month follow-up

| Characteristic | Incomplete 6-month questionnaire  N = 158 (%) | Complete 6-month questionnaire  N = 148 (%) | *P** |
| --- | --- | --- | --- |
| Age, years |  |  | 0.5 |
| 65-75 | 74 (47) | 79 (53) |  |
| 75-85 | 59 (37) | 48 (32) |  |
| >85 | 25 (16) | 21 (14) |  |
| Female | 69 (44) | 69 (47) | 0.6 |
| Married | 106 (67) | 104 (70) | 0.6 |
| Race |  |  | < 0.001 |
| White | 99 (63) | 130 (89) |  |
| Asian | 25 (16) | 2 (1) |  |
| Other or unknown | 34 (22) | 16 (11) |  |
| Pre-admission living situation |  |  | 0.09 |
| Home without assistance | 109 (69) | 118 (80) |  |
| Home with assistance | 39 (25) | 25 (17) |  |
| Other | 10 (6) | 5 (3) |  |
| Charlson Comorbidity Index, median (IQR) | 1 (0-2) | 1 (0-2) | 0.2 ^†^ |
| Abnormal hemoglobin at admission |  |  |  |
| Men (<140g/L or >185g/L) | 42 (27) | 31 (21) | 0.3 |
| Women (<123g/L or >165g/L) | 27 (17) | 19 (13) | 0.2 |
| Total admission medications, median (IQR) | 4 (2−7) | 4 (2−7) | 0.3 ^†^ |
| Clinical Frailty Score, mean ± SD | 3.7 ± 1.3 | 3.1 ± 1.3 | < 0.001 |
| ASA Physical Status Class | 2.7 ± 0.8 | 2.7 ± 0.7 | 0.9 |
| Surgery Type |  |  | 0.09 |
| Closed appendectomy or cholecystectomy | 44 (28) | 42 (28) |  |
| Open appendectomy or cholecystectomy | 9 (6) | 14 (10) |  |
| Hernia | 16 (10) | 26 (18) |  |
| Small intestine | 47 (30) | 39 (26) |  |
| Colon | 23 (15) | 20 (14) |  |
| Other | 19 (12) | 7 (5) |  |
| Ostomy creation procedure | 14 (9) | 14 (10) | 0.9 |
| Delayed post-operative mobilization | 35 (22) | 39 (26) | 0.4 |
| Length of stay | 9 (6−14) | 8 (6−13) | 0.09 ^†^ |
| Notes: ASA=American Society of Anesthesiologists; SD=standard deviation, IQR=interquartile range.  **X*^2^ or Fischer’s exact tests for categorical and *t*-test for continuous variables unless otherwise specified.  †Mann-Whitney test. | | | |
