# Supplementary material for: The impact of delayed mobilization on post-discharge outcomes after emergency abdominal surgery: A prospective cohort study in older patients
Source: PLoS One. 2020 Nov 6;15(11):e0241554. doi: 10.1371/journal.pone.0241554 (PMC7647086; doi:10.1371/journal.pone.0241554)
Supplement: S2 Table — (DOCX) [file pone.0241554.s002.docx]

S2 Table: Changes in health-related quality-of-life from 5 weeks to 6 months after surgical discharge

| Outcome | Time of mobilization, mean difference from 5 weeks to 6 months ± SD | | Comparison over time,† difference of mean difference (p-value) |
| --- | --- | --- | --- |
|  | Delayed | Early | Delayed *vs*. early |
| EQ-5D | 0.10 ± 0.17, n = 29 | 0.02 ± 0.16, n = 91 | 0.08 (0.03)* |
| EQ-VAS | 7.1 ± 16.7, n = 29 | -1.9 ± 17.5, n = 94 | 9.0 (0.02) |
| SF-12, physical | 4.3 ± 9.2, n = 28 | 0.0 ± 6.5, n = 93 | 4.3 (0.007) |
| SF-12, mental | 1.5 ± 10.1, n = 28 | 1.6 ± 7.4, n = 92 | 0.1 (>0.99) |
| Notes: EQ-5D=EurolQol five dimension index score; EQ-VAS=visual analogue scale; SF-12=12-item Short-form Health Survey; SD=standard deviation  *Minimal clinically important difference (EQ-index=0.03; EQ-VAS=10.0; SF-12=5.0)  †From 5 weeks to 6 months after discharge | | | |
