# Supplementary material for: The impact of delayed mobilization on post-discharge outcomes after emergency abdominal surgery: A prospective cohort study in older patients
Source: PLoS One. 2020 Nov 6;15(11):e0241554. doi: 10.1371/journal.pone.0241554 (PMC7647086; doi:10.1371/journal.pone.0241554)
Supplement: S3 Table — (DOCX) [file pone.0241554.s003.docx]

S3 Table: Delayed mobilization and readmission or death, stratified by frailty status

|  |  | Readmitted or died | | | Relationship of delayed mobilization and outcome, Multivariable logistic regression | |
| --- | --- | --- | --- | --- | --- | --- |
|  |  | Yes | No | % Yes | aOR (95 % CI) | *P* |
| 30 days |  | N=41 | N=265 |  |  |  |
| Vulnerable-to-moderately-frail | Delayed | 15 | 42 | 26 | 3.39 (1.22-9.38) | 0.02 |
|  | Early | 11 | 65 | 14 |  |  |
| Very-fit-to-managing-well | Delayed | 4 | 20 | 17 | 2.50 (0.68-9.14) | 0.2 |
|  | Early | 11 | 146 | 7 |  |  |
| 6 months |  | N=102 | N=204 |  |  |  |
| Vulnerable-to-moderately-frail | Delayed | 31 | 26 | 54 | 2.74 (1.08-6.93) | 0.03 |
|  | Early | 30 | 46 | 39 |  |  |
| Very-fit-to-managing-well | Delayed | 7 | 17 | 29 | 1.45 (0.55-3.95) | 0.5 |
|  | Early | 34 | 123 | 22 |  |  |
| Notes: CI=confidence interval; aOR=adjusted odds ratio.  Models were adjusted for age, sex, comorbidities, total medications, abnormal hemoglobin, and time in surgery. | | | | | | |
