# Supplementary material for: The impact of delayed mobilization on post-discharge outcomes after emergency abdominal surgery: A prospective cohort study in older patients
Source: PLoS One. 2020 Nov 6;15(11):e0241554. doi: 10.1371/journal.pone.0241554 (PMC7647086; doi:10.1371/journal.pone.0241554)
Supplement: S4 Table — (DOCX) [file pone.0241554.s004.docx]

S4 Table: Time to mobilization and readmission or death

| Hours to mobilization (total N) | 30-day Readmission/Death  N (%) | p-value^a^ | 6-month Readmission/Death  N (%) | p-value^a^ |
| --- | --- | --- | --- | --- |
| 0-12 (99) | 10 (10) |  | 27 (27) |  |
| 12-23 (102) | 10 (10) |  | 30 (29) |  |
| 24-35 (31) | 2 (7) |  | 7 (23) |  |
| 36-48 (27) | 8 (30) |  | 12 (44) |  |
| ≥48 (47) | 11 (23) | 0.009 | 26 (55) | 0.003 |
| ^a^Chi-2 test. | | | | |
